# Effects of a visual‐feedback LED pacing system in middle-distance pool freestyle swimming

**DOI:** 10.3389/fbioe.2025.1679588

**Published:** 2025-10-21

**Authors:** Zulin Chen, Lihan Lin, Yuqi He, Yikun Zheng, Weiwei Zhang, Dingtao Yan, Shumin Luo, Huangtao Chen, Hongmiao Chen

**Affiliations:** ^1^ College of Physical Education, Huaqiao University, Quanzhou, China; ^2^ Research Center for Sports and Health Sciences, Huaqiao University, Quanzhou, China; ^3^ Provincial University Key Laboratory of Sport and Health Science, School of Physical Education and Sport Science, Fujian Normal University, Fuzhou, China; ^4^ Department of Life Science, The University of Tokyo, Tokyo, Japan; ^5^ College of Engineering, Huaqiao University, Quanzhou, China

**Keywords:** LED pacing system, freestyle swimming, visual feedback, segmental pacing, blood lactate, heart rate

## Abstract

**Objective:**

This study examined the effects of an intelligent LED pacing system with visual feedback on training performance and physiological responses in middle-distance freestyle swimming.

**Methods:**

Twelve high-level swimmers completed two 200 m freestyle trials in a self-controlled design: first a self-paced swim, followed 24 h later by an LED-paced swim. The LED system (Chaser-S1, Huaqiao University) converted individualized target times from each swimmer’s personal best into underwater light signals, providing real-time feedback. Trials were conducted in a 50 m indoor pool under standardized conditions. Each race was timed by three referees with synchronized stopwatches, heart rate was monitored with a Polar H10 under the swim cap, and fingertip blood lactate was sampled 1 min before and after each trial. Recorded outcomes included entire and segmental times, coefficient of variation (CV) of split time, blood lactate change (Δ mmol·L^−1^), heart rate range, and normalized HR range (HR CV). Normality was tested (Shapiro–Wilk), and paired-sample t-tests with Bonferroni adjustment (adjusted α = 0.01) were applied. Results included t values, *P* values, 95% confidence intervals, and effect sizes (Cohen’s *d*).

**Results:**

LED pacing showed no significant differences in entire 200 m time or split times compared with self-pacing (entire: 150.21 ± 18.44 vs. 153.78 ± 20.26 s, *d* = 0.185; *P* = 0.014, not significant after Bonferroni adjustment). Pacing stability, assessed by CV of split times, showed no significant difference between LED pacing (8.49% ± 2.82%) and self-pacing (8.48% ± 2.56%) conditions (*P* = 0.981, *d* = 0.004). Physiologically, LED pacing lowered blood lactate accumulation (Δ lactate: 7.18 ± 1.61 vs. 8.63 ± 1.19 mmol L^−1^, *P* = 0.028, *d* = 1.019), smaller heart rate fluctuations (overall range: 45.50 ± 7.40 vs. 53.67 ± 7.75 bpm, *P* < 0.001, *d* = 1.078), and reduced HR CV across all segments (overall HR CV: 27.18% ± 4.46% vs. 32.51% ± 5.07, *P* < 0.001, *d* = 1.117).

**Conclusion:**

In highly trained swimmers, LED pacing exerted negligible effects on pacing consistency and race time but was associated with reduced post-exercise blood lactate and heart rate fluctuations, indicating potential utility for training load management rather than immediate performance enhancement.

## 1 Introduction

The competitive performance in middle-distance freestyle swimming is determined not only by an athlete’s physical capacity and technical proficiency but is also highly dependent on scientific pacing strategies and precise rhythm control (Abbiss and Laursen, 2008). Numerous studies have demonstrated that rational energy distribution, maintaining a stable swimming velocity, and executing a strong finish in the middle and latter stages are core factors influencing final performance outcomes (Lara and Del Coso, 2021; Fang et al., 2024). However, in both training and competition, athletes frequently encounter pacing errors, for example, in the 200 m freestyle, athletes often display a fast-start followed by mid-race deceleration with only a limited end-spurt (a parabolic/positive pacing profile), or show cumulative drift from planned splits that results in progressive velocity decay across segments. These pacing errors can stem from subjective misjudgment of their own velocity as well as limitations inherent in traditional training methods that lack real-time, quantifiable pacing feedback mechanisms (Altavilla et al., 2018).

In recent years, rapid advancements in sports technology have introduced transformative tools for competitive training. Intelligent LED pacing systems, for example, convert predetermined target speeds into intuitive visual cues, providing athletes with real-time, precise external rhythm guidance (Altavilla et al., 2018; Szczepan and Zatoń, 2017). Related research indicates that such external rhythmic stimulation can reinforce athletes’ sense of pacing, reduce fluctuations caused by subjective judgment, and minimize pacing errors and unnecessary energy expenditure (Morais et al., 2022). In track and field, comparable LED pacing lights have been successfully implemented in international competitions to assist athletes in breaking records (Grannetia and Hoogkamer, 2024). In aquatic environments, LED pacing has unique adaptive advantages: light signals remain clearly visible and stable underwater, and the fixed lanes and forward-facing vision of swimmers make visual guidance especially effective compared with land-based settings. Beyond pacing execution, such external cues may reduce distribution errors, leading to more economical energy use. These adjustments are expected to manifest in physiological outcomes. Consistent with these adaptive features, previous studies in swimming have shown that LED visual feedback during 50–150 m freestyle can enhance segmental pacing stability and improve the accuracy of speed control (Szczepan and Zatoń, 2017; Gonjo et al., 2021). Although these studies did not directly measure fatigue or physical output decline, the improved precision in pacing suggests a potential to support more economical energy distribution and delay performance deterioration.

Nevertheless, empirical research on the application of LED pacing systems in middle-distance swimming remains insufficient, particularly with regard to systematic analysis of segmental pacing dynamics, pacing stability, and concomitant physiological changes (Gonjo et al., 2021; Neuloh et al., 2024). Unlike sprint (<100 m) or long-distance (>400 m) swimming events, the 200 m freestyle requires a unique combination of anaerobic and aerobic metabolism, speed endurance, and precise pacing strategies (Moser et al., 2021; Demarie et al., 2022; Fernandes et al., 2024). These distinctive physiological and technical demands make the 200 m event an ideal model for evaluating the application of LED pacing systems. Furthermore, the integration of pacing systems with heart rate and physiological load monitoring is still developing, presenting considerable opportunities to advance the scientific and individualized nature of swim training (Sixsmith et al., 2023).

In this study, we define segmental pacing dynamics as the changes in velocity across consecutive race segments and pacing stability as the overall uniformity of pacing, measured by the coefficient of variation (CV) of split times (Fang et al., 2024; Neuloh et al., 2024). These indicators are complementary: dynamics capture where LED guidance exerts influence within a race, while stability reflects the consistency of pacing across the entire 200 m. In addition, concomitant changes in physiological load, such as heart rate fluctuations and post-exercise blood lactate responses, provide further insight into the internal demands elicited by pacing interventions. Given that sustaining mid-race speed is often decisive in middle-distance swimming, analyzing both provides a clearer understanding of the potential benefits of LED pacing systems (Gonjo et al., 2021).

Against this background, the present study focuses on 200 m middle-distance freestyle swimming, employing a pre-post self-controlled design to systematically evaluate the effects of an intelligent LED pacing system on event performance, segmental pacing, and pacing stability. Beyond overall performance and pacing outcomes, the study also considers physiological load indicators such as heart rate and blood lactate in order to capture the internal demands of LED-guided swimming. This research conducts an in-depth analysis of segmental pacing patterns and inter-individual differences, aiming to provide empirical support for the intelligent and refined management of swim training and to offer theoretical and practical insights for the future integration of intelligent pacing systems with multi-parameter monitoring.

Based on previous findings in related endurance sports, we hypothesize that real-time visual feedback from an LED pacing system may facilitate improved performance in middle-distance swimming by promoting more efficient energy distribution during the race and reducing unnecessary physiological strain, particularly by limiting metabolic stress (excessive lactate accumulation) and neuromuscular fatigue (heart rate instability). Specifically, we anticipate that such a system could help swimmers achieve faster split times in certain race segments, limit the accumulation of blood lactate, and maintain more stable heart rate responses under high-intensity conditions.

## 2 Materials and methods

### 2.1 Participants

Twelve high-level swimmers (national second-class freestyle athletes; 6 males and 6 females; training experience ≥5 years) from the Huaqiao University swimming team participated in this study. All athletes held the title of “National Second-Class Athlete” according to the *Chinese Athlete Technical Grade Standard* issued by the General Administration of Sport of China. Inclusion criteria were: (Abbiss and Laursen, 2008): at least 5 years of systematic professional training; (Lara and Del Coso, 2021); good overall health without major musculoskeletal or cardiopulmonary disorders. Exclusion criteria were: (Abbiss and Laursen, 2008): injury or surgery within the past 3 months; (Lara and Del Coso, 2021); use of medications that could affect cardiovascular or metabolic function. Baseline demographic and performance characteristics, including age, sex, height, body mass, BMI, training experience, and personal best 200 m freestyle pool time, were collected and are presented in Supplementary Table S1. The sample size (n = 12) was determined *a priori* using power analysis (G*Power 3.1) for a paired t-test with α = 0.05, power = 0.80, and an expected large effect size (Cohen’s d = 0.8), in line with established recommendations (Cohen, 1988), indicating sufficient power to detect significant effects (Cohen, 2013).

### 2.2 Instruments and equipment

**FIGURE 1:**
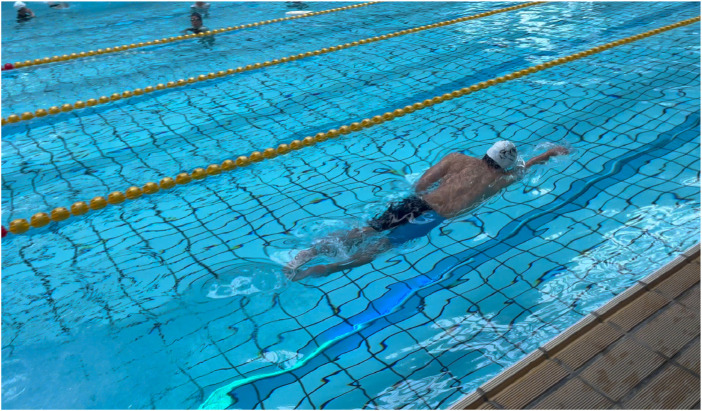
Images of the Chaser-S1 system.

**FIGURE 2:**
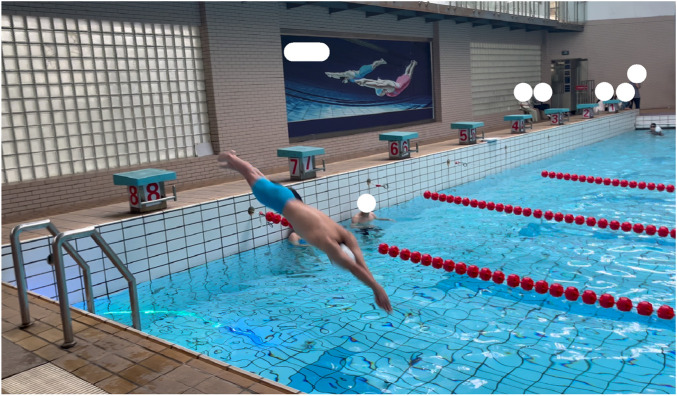
Application of the Chaser-S1 system in a real swimming training environment.

**FIGURE 3:**
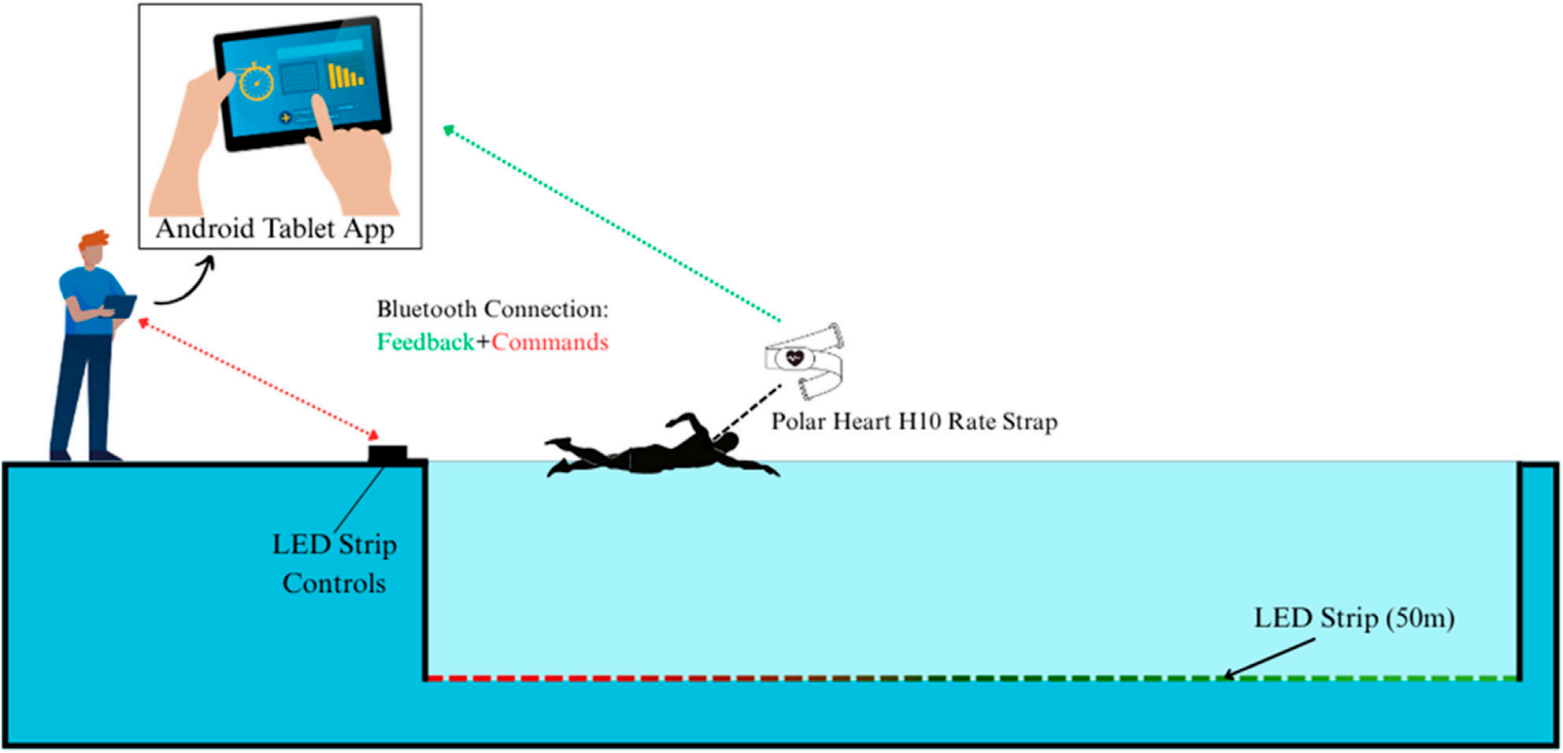
Schematic diagram of the intelligent LED pacing system used for real-time visual feedback in swimming trials.

(1) Intelligent LED pacing system (Chaser-S1, developed by the Center for Sport and Health Sciences, Huaqiao University): This system operates on an Android platform, with Chaser-S1 software capable of intelligently controlling the speed of a 50 m LED strip. It utilizes embedded technology, Bluetooth communication, optoelectronic technology, and Flutter technology. The system is operated via a mobile device app, which allows users to set the pacing speed based on their needs. Through Bluetooth communication, the system controls the light flow speed of the LED strip, enabling preset pace adjustments for intervals of at least 10 m with an accuracy of ≤0.01 s. Building upon previously validated pool-based LED pacing devices (Szczepan and Zatoń, 2017; Hexia et al., 2016), the Chaser-S1 incorporates incremental improvements aimed at enhancing control stability and operational precision. Images of the Chaser-S1 system and its practical implementation are shown in Figures 1, 2. (2) Casio stopwatch (CASIO HS70W, Casio, Japan): Used to record 50 m split times and total race time with a precision of 0.001 s (3) Portable blood lactate analyzer (Lactate Plus, Nova Biomedical, United States): This handheld analyzer measures blood lactate concentration in approximately one second, with an accuracy of ±0.2 mmol L^−1^, using a micro-blood sample (∼5 μL) via electrochemical sensor technology for real-time assessment. (4) Polar H10 heart rate monitor (Polar, Finland): The Polar H10 employs ECG electrodes to detect cardiac activity. Although the device is capable of high-frequency ECG sampling (up to 1000 Hz), in exercise settings it typically provides beat-to-beat–derived heart rate values at 1-s intervals, which were used in this study for segmental and overall analyses. Data were transmitted via Bluetooth and exported using the BHT Team APP (version V1.2.3). The system’s specific working process is illustrated in Figure 3.

### 2.3 Experimental procedures

#### 2.3.1 Quality control

A pre-post self-controlled experimental design was employed in the 200 m freestyle event to assess the practical impact of the intelligent LED pacing system on training outcomes. Baseline demographic and performance data were collected prior to testing, including measured variables (age, height, body mass, BMI) and self-reported variables (training experience and personal best 200 m freestyle pool time), in order to confirm the competitive level and homogeneity of the sample. All tests were conducted in the same indoor heated pool (50 m length, 21 m width, 1.7–2.1 m depth, water temperature 26 °C ± 0.5 °C) during the same time window (14:00–18:00). Participants’ diet, sleep, and training load were standardized for 24 h prior to testing to eliminate potential confounding variables and ensure environmental consistency. Given that the participants were elite swimmers accustomed to daily high-intensity training, a 24-h interval between trials was considered acceptable and broadly consistent with their routine recovery patterns (Faghy et al., 2019; Nikseresht et al., 2017; Sládečková et al., 2024).

Each lane was timed by three independent national-level swimming referees using synchronized handheld stopwatches (CASIO HS-70W), with the average taken and rounded to 0.01 s. Although an electronic touch-pad system is considered the gold standard, such equipment was not available at the facility; therefore, the use of experienced referees minimized potential error.

Blood lactate measurements were performed by professionals using fingertip sampling according to standard operating procedures; analyzers were calibrated daily, and all samples were analyzed within 5 min of collection. Capillary blood was obtained 1 min post-exercise to ensure consistency in the pool environment; this protocol may underestimate individual peak values, but the uniform sampling strategy allowed valid comparisons between conditions.

The LED pacing system was managed by dedicated personnel to ensure stable and accurate operation according to preset protocols. Heart rate was monitored by experienced operators, who secured the Polar H10 sensor under the swim cap on the swimmer’s forehead using an elastic band and medical-grade tape to maintain continuous skin contact. A series of preliminary pilot tests demonstrated that forehead placement under the swim cap produced fewer dropouts and more stable recordings than chest placement, which often suffered from electrode displacement in water (Olstad et al., 2020; Bartolomeu et al., 2025). In addition, the swim cap acted as a barrier that reduced water interference at the electrode-skin interface. As only 1-Hz heart rate values were required to calculate heart rate range and coefficient-based variability, rather than RR-interval HRV, this configuration provided uninterrupted and stable recordings. The sensor was wirelessly connected to a tablet via Bluetooth, allowing for real-time monitoring of heart rate data.

Heart rate recording was started simultaneously with the race start signal, and split times from three timekeepers were used to segment the HR data. A sensitivity check with ±1 s boundary shifts showed consistent results, indicating that potential latency had no impact on data analysis. This process ensured continuous and accurate data collection throughout the test. The LED pacing system was managed by technical staff who were only responsible for device operation and had no role in outcome measurement or data analysis.

Blinding procedures were partially implemented: lactate assessors recorded samples by athlete ID without knowledge of pacing condition; heart rate data were exported and processed by operators who were not informed of group allocation. Full blinding of timekeepers was not feasible because the LED pacing lights were visible, but they were instructed to record independently without access to prior results.

#### 2.3.2 Experimental protocol

(1) Step one: On the first day, participants completed a maximal 200 m freestyle sprint without LED pacing. Each 50 m split and the total race time were recorded in detail. Blood lactate samples were collected within 1 min before and after the test to assess baseline and post-exercise lactate levels. Heart rate was monitored throughout the test. (2) Step two: Based on their split times and subjective feedback from the first test, each swimmer adjusted their pacing for subsequent segments to develop an individualized optimal pacing strategy, with the total predicted race time set to match their personal best performance in the 200 m freestyle. (3) Step three: 24 h after the initial test, participants completed a second maximal 200 m freestyle trial, this time with guidance from the intelligent LED pacing system. All split and total times were again recorded, and blood lactate was sampled within 1 min before and after the test to allow comparison of metabolic responses under different pacing strategies. Heart rate was again monitored using the same method as in step one throughout the test.

### 2.4 Statistical analysis

All statistical analyses were conducted using SPSS 26.0 software. Descriptive statistics were used to summarize all performance, heart rate, and blood lactate variables. The CV for split times and the normalized HR range was calculated and reported as CV%. All data, including baseline demographic variables (age, sex, height, body mass, BMI, training experience, and personal best 200 m freestyle pool time) as well as performance times, heart rate, blood lactate values, and CV% for split times and heart rate, were initially treated as continuous variables where applicable. Normality of continuous variables was assessed using the Shapiro–Wilk test. All variables other than age were normally distributed (P > 0.05) and are expressed as means ± SD; age, which did not follow a normal distribution, is expressed as median (IQR). (1) Race performance, including split times for each 50 m segment and total 200 m race time, was summarized, and the CV was calculated for split times as well as for the total race time. For heart rate, the range (max–min) and the CV were calculated for each 50 m segment and the entire 200 m swim. Blood lactate values were reported as pre-exercise and post-exercise values for the entire 200 m trial, and the change in lactate (Δ lactate) was calculated as the mean difference between pre- and post-test values. All data were calculated and presented separately for the two pacing conditions (Self-paced and LED Pacing). (2) Time and normalized HR range were assessed using the CV. For split time variability, the CV of time was calculated using the formula:

$${\text{CV}}_{\text{split}}=\frac{\text{SD}\;\text{of}\;\text{the}\;4\;\text{split}\;\text{times}}{\text{mean}\;\text{of}\;4\;\text{split}\;\text{times}}\times 100\%$$

For normalized HR range, a CV was calculated using the following simplified range-based formula:

$$\text{HR CV}=\frac{\max\text{HR}‐\min\text{HR}}{\text{mean}\;\text{HR}}\times 100\%$$

This formula quantifies the relative amplitude of heart rate changes during each race segment, providing a practical and interpretable indicator of pacing steadiness in high-intensity swimming. Compared with conventional RR-interval HRV metrics, it offers greater feasibility in aquatic settings while still capturing meaningful cardiovascular fluctuations. Although this normalized HR range is highly correlated with the absolute HR range, it was retained because its relative expression reduces inter-individual bias from differing baseline HR levels, thereby facilitating fairer comparisons across athletes and conditions.

(3) Paired-sample t-tests were employed to compare race times, heart rate, and blood lactate changes under the two pacing conditions (Self-paced and LED pacing), with adjustments made for multiple comparisons. The paired-sample t-test was used to compare: (a) Split times for each 50 m segment (0–50 m, 50–100 m, 100–150 m, and 150–200 m) and the total 200 m completion time, (b) Time variability (CV%) for each 50 m segment and the overall 200 m race time, (c) Blood lactate changes (Δ lactate) before and after the exercise test, (d) Heart rate range (max–min) for each 50 m segment and the overall 200 m swim, (e) Heart rate variability (CV%) for each 50 m segment and the overall 200 m swim. For each comparison, t-values (*t*), P-values (*P*), 95% confidence intervals (95% CI), and Cohen’s d (ES, *d*) were reported, (4) To control for multiple comparisons, Bonferroni correction was applied within each outcome domain. For split times, HR range, and HR CV, five comparisons were conducted (corresponding to four 50-m segments and the overall 200-m race), so the adjusted significance threshold was set at α = 0.01 (0.05/5). For single comparisons (blood lactate, pacing stability CV%), the original α = 0.05 was retained. (5) Effect sizes were calculated using Cohen’s *d*, with thresholds for small, medium, and large effect sizes defined as 0.20, 0.50, and 0.80, respectively.

### 2.5 Ethical approval and reporting standards

This study was conducted in accordance with the Declaration of Helsinki. The study protocol was reviewed and approved by the Medical Ethics Committee of Huaqiao University School of Medicine (Approval No. M2025026). All participants were fully informed of the study procedures, potential risks, and benefits prior to enrollment, and each provided written informed consent. Personal data and performance results were anonymized and treated confidentially, and all data were used exclusively for research purposes. This study followed the TREND (Transparent Reporting of Evaluations with Nonrandomized Designs) checklist to ensure comprehensive and transparent reporting.

## 3 Results

### 3.1 Baseline characteristics

Baseline demographic and performance characteristics of the participants are presented in Table 1. The median age of the participants was 20.00 years (IQR: 20.00–20.25). On average, the participants were 1.75 ± 0.07 m tall, weighed 67.42 ± 6.26 kg, and had a BMI of 22.09 ± 1.56 kg m^−2^. Their training background was 14.00 ± 2.63 years, and the mean personal best 200 m freestyle performance was 137.76 ± 8.96 s. The sample was relatively homogeneous in demographic and performance characteristics (Table 1).

**TABLE 1:** Baseline demographic and performance characteristics of participants (n = 12).

Variable	Male (n = 6)	Female (n = 6)	Total (n = 12)
Age (years)	20.50 (20.00, 21.75)	20.00 (20.00, 20.00)	20.00 (20.00, 20.25)
Height (m)	1.78 ± 0.06	1.71 ± 0.06	1.75 ± 0.07
Body mass (kg)	71.26 ± 4.75	63.57 ± 5.32	67.42 ± 6.26
BMI (kg/m^2^)	22.58 ± 1.83	21.60 ± 1.20	22.09 ± 1.56
Training experience (years)	13.33 ± 3.67	14.67 ± 0.82	14.00 ± 2.63
Personal best 200 m freestyle pool time (s)	133.86 ± 8.58	141.66 ± 8.14	137.76 ± 8.96

### 3.2 Race split times and total time across pacing modes

The overall 200 m completion time was lower under the LED pacing condition compared to the self-paced condition (150.21 ± 18.44 s vs. 153.78 ± 20.26 s, raw *P* = 0.014, Cohen’s *d* = 0.185). Although this difference reached nominal significance, it did not survive Bonferroni correction (adjusted α = 0.01), and the effect size indicated only a small magnitude of improvement.

For the 50 m splits, raw analyses indicated shorter times in the middle segments (50–100 m and 100–150 m) under LED pacing (37.79 ± 4.51 s vs. 38.54 ± 4.53 s, *P* = 0.018, Cohen’s *d* = 0.166; 39.33 ± 4.96 s vs. 40.50 ± 5.63 s, *P* = 0.014, Cohen’s *d* = 0.220). However, neither difference remained significant after Bonferroni correction, and both effect sizes were small.

**FIGURE 4:**
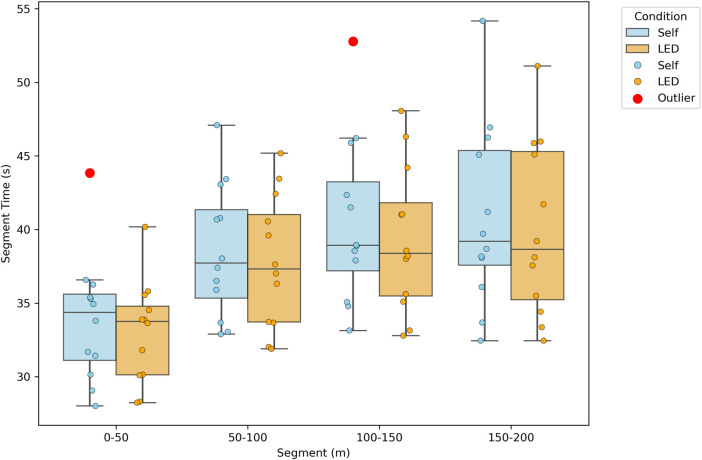
Segmental performance times across race sections under self-paced and LED pacing conditions.

By contrast, the 0–50 m and 150–200 m segments showed no statistically significant differences between pacing conditions in either the raw or corrected analyses (*P* = 0.056 and 0.071, Cohen’s *d* = 0.223 and 0.139, respectively). These results further indicate that, after correction for multiple comparisons, LED pacing did not meaningfully influence race completion time or segmental performance (Table 2; Figure 4).

**TABLE 2:** Split times (s) for 200 m pool freestyle under self-paced and LED-paced conditions (n = 12).

Segment	Self-paced	LED pacing	*t*	*P*	95% CI	ES, *d*
0–50 m	33.87 ± 4.26	33.00 ± 3.48	2.133	0.056	−0.028–1.761	0.223
50–100 m	38.54 ± 4.53	37.79 ± 4.51	2.782	0.018	0.157–1.345	0.166
100–150 m	40.50 ± 5.63	39.33 ± 4.96	2.910	0.014	0.285–2.052	0.220
150–200 m	40.88 ± 6.23	40.03 ± 5.92	1.998	0.071	−0.086–1.772	0.139
0–200 m (entire segment)	153.78 ± 20.26	150.21 ± 18.44	2.911	0.014	0.872–6.276	0.185

### 3.3 CV of split time across pacing modes

Pacing stability, assessed by the CV of split times, showed no significant difference between the LED-paced and self-paced conditions (8.49% ± 2.82% vs. 8.48% ± 2.56%, *P* = 0.981, Cohen’s *d* = 0.004). This negligible effect size indicates that LED pacing did not improve or diminish split time consistency compared to traditional self-pacing. These results suggest that LED pacing had no substantial impact on pacing stability, as reflected by the variability in segmental split times across the 200 m trial (Table 3).

**TABLE 3:** Comparison of split time CV (%) between self-pacing and LED pacing conditions (n = 12).

Pacing condition	CV%	*t*	*P*	95% CI	ES, *d*
Self-paced	8.48 ± 2.56	−0.024	0.981	−1.011–0.989	0.004
LED Pacing	8.49 ± 2.82				

### 3.4 Change in blood lactate concentration before and after the 200 m swim across pacing modes

**FIGURE 5:**
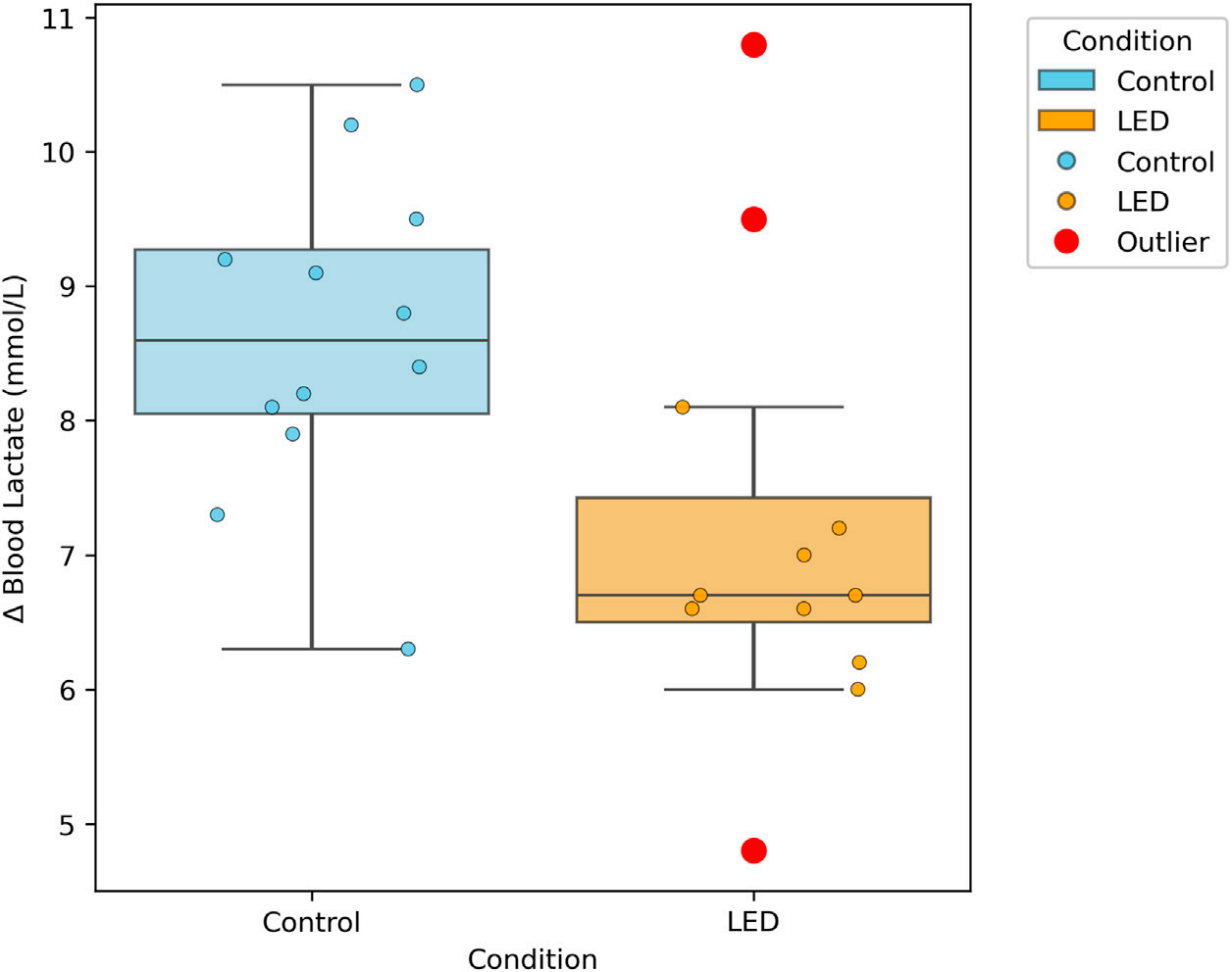
Changes in blood lactate concentrations from pre-to post-exercise under self-paced and LED pacing conditions.

The change in blood lactate concentration (Δ lactate, pre-to post-exercise) was significantly different between the two pacing conditions (*P* ≤ 0.05). Specifically, the post-exercise increase in lactate was greater in the self-paced trials (Δ = 8.63 ± 1.19 mmol L^−1^) than in the LED-paced trials (Δ = 7.18 ± 1.61 mmol L^−1^; *P* = 0.028, Cohen’s *d* = 1.019). Here, Cohen’s d denotes a large effect size, reflecting a substantial difference in blood lactate response between conditions: LED pacing elicited a notably smaller post-exercise lactate increase than self-pacing (Table 4; Figure 5). Taken together, the blood lactate difference is a relatively meaningful physiological finding of this study.

**TABLE 4:** Blood-lactate concentration (mmol ⋅ L−^1^) before and after the 200 m trial under two pacing conditions (n = 12).

Pacing condition	Pre-exercise lactate	Post-exercise lactate	*Δ lactate	*t* (Δ)	*P* (Δ)	95% CI (Δ)	ES, *d* (Δ)
Self-paced	5.38 ± 1.20	14.00 ± 1.99	−8.63 ± 1.19	2.534	0.028	0.189∼2.694	1.019
LED Pacing	4.84 ± 2.13	12.03 ± 2.63	−7.18 ± 1.61				

*Δ Denotes the difference between two values; in this context, Δ specifically refers to the change in lactate concentration before and after exercise.

### 3.5 Heart rate range and dynamics across pacing modes

The maximum–minimum heart rate range during the 200 m swim was significantly smaller after Bonferroni correction under LED pacing compared to self-pacing for every 50 m segment and for the overall trial (all paired *P* ≤ 0.01). For example, in the 50–100 m segment, the heart rate range was 9.50 ± 1.31 beats·min^-1^ with LED pacing versus 13.42 ± 1.93 beats·min^−1^ with self-paced swimming (*P* < 0.001, Cohen’s *d* = 2.373), representing a very large effect size and indicating a marked reduction in heart rate fluctuation. At the 0–50 m segment, the range was 9.08 ± 1.31 bpm (LED) versus 11.00 ± 1.86 bpm (self-paced, *P* = 0.002, Cohen’s *d* = 1.192, large effect size), further supporting a substantial difference. Significant reductions in heart rate range with LED pacing were also observed at 100–150 m (12.50 ± 2.71 vs. 13.33 ± 2.96 bpm, *P* < 0.001, Cohen’s *d* = 0.293, small effect size) and 150–200 m (14.00 ± 2.63 vs. 15.92 ± 2.97 bpm, p < 0.001, Cohen’s d = 0.684, medium effect size). Over the entire 200 m, the total heart rate range was lower in the LED-paced condition (45.50 ± 7.40 bpm) than in the self-paced condition (53.67 ± 7.75 bpm; *P* < 0.001, Cohen’s *d* = 1.078, large effect size).

**FIGURE 6:**
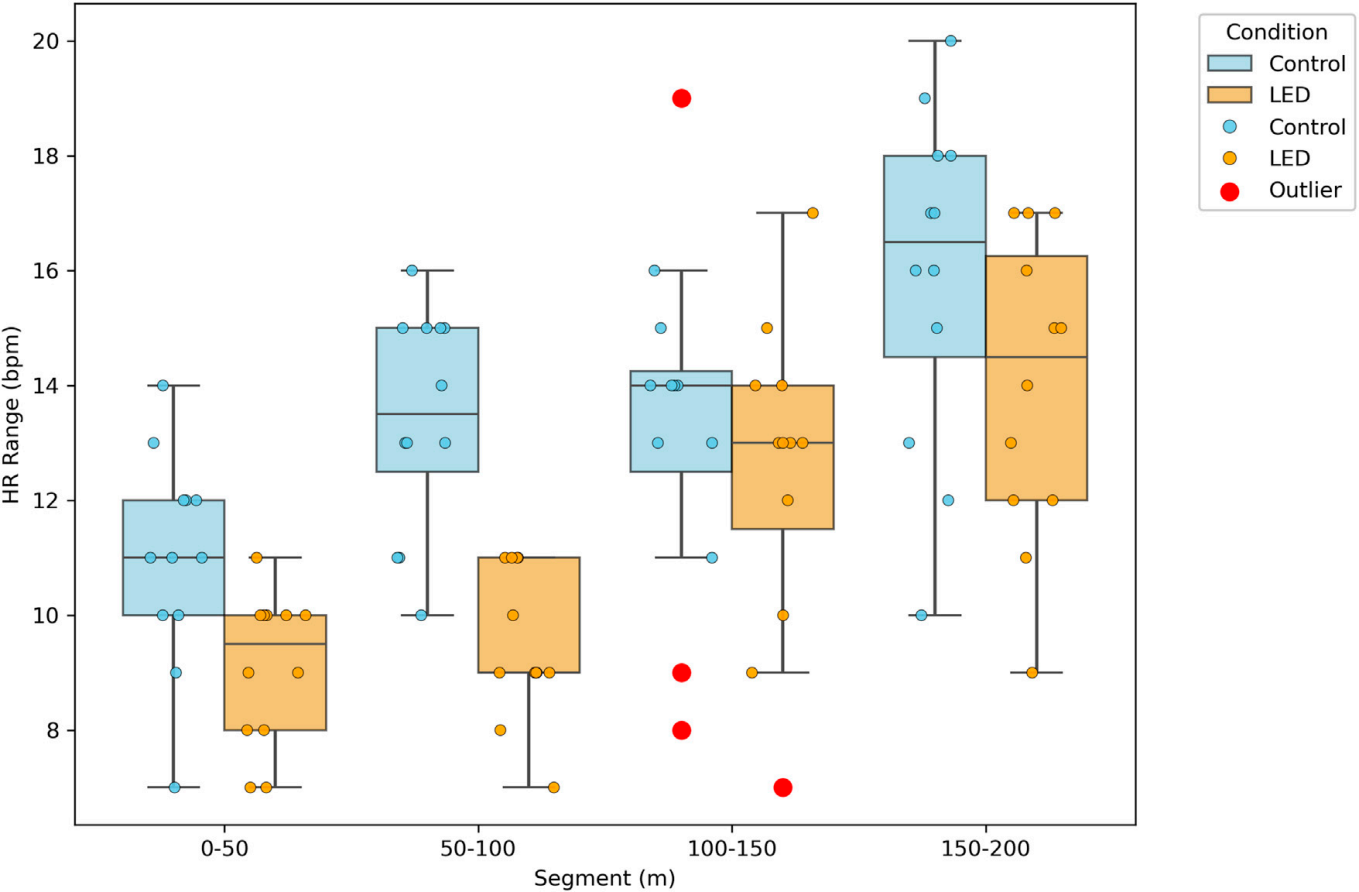
Segmental heart rate range across race sections under self-paced and LED pacing conditions.

These results indicate that LED pacing led to a substantial reduction in heart rate fluctuation, particularly in the early (0–100 m) and overall segments, with less pronounced but still significant effects in the latter (100–200 m) segments of the swim (Table 5; Figure 6).

**TABLE 5:** Comparison of HR range (Max–Min) between control and LED pacing conditions across segments of a 200-m pool freestyle swim (n = 12).

Segment	Self-paced	LED pacing	*t*	*P*	95% CI	ES, *d*
0–50 m	11.00 ± 1.86	9.08 ± 1.31	3.960	0.002	0.851∼2.982	1.192
50–100 m	13.42 ± 1.93	9.50 ± 1.31	10.346	<0.001	3.083∼4.750	2.373
100–150 m	13.33 ± 2.96	12.50 ± 2.71	5.000	<0.001	0.467∼1.200	0.293
150–200 m	15.92 ± 2.97	14.00 ± 2.63	5.702	<0.001	1.177∼2.657	0.684
0–200 m (entire segment)	53.67 ± 7.75	45.50 ± 7.40	9.494	<0.001	6.273∼10.060	1.078

### 3.6 Normalized HR range across pacing modes

Correlation analysis showed that HR range and HR CV were highly correlated across all race segments (*r* > 0.90, all *P* < 0.001). Nevertheless, both indices are reported here because they reflect complementary aspects of cardiovascular load: the absolute amplitude (HR range) and the relative steadiness (HR CV).

Heart rate variability, expressed as the CV of heart rate, was significantly lower after Bonferroni correction in the LED-paced trials compared with self-paced trials across all segments of the swim (p ≤ 0.01 for all comparisons). The largest reduction occurred in the 50–100 m segment, where the HR CV decreased from 8.58% ± 1.44 (self-paced) to 5.94% ± 0.89 (LED-paced, *P* < 0.001, Cohen’s *d* = 2.204), corresponding to a very large effect size. This finding denotes an exceptionally strong difference between conditions, with LED pacing providing a more stable heart rate during this phase.

Substantial reductions were also observed in the 0–50 m (7.60% ± 1.30% vs. 6.05% ± 0.98, *P* < 0.001, Cohen’s *d* = 1.345, large effect size) and 150–200 m (8.60% ± 1.55% vs. 7.57% ± 1.33, *P* < 0.001, Cohen’s *d* = 0.715, medium effect size) segments. Even the 100–150 m segment showed a smaller yet statistically significant decrease in HR CV (7.85% ± 1.81% vs. 7.29% ± 1.57, *P* = 0.001, Cohen’s *d* = 0.328, small effect size). Across the entire 200 m trial, the mean HR CV was lower under LED pacing (27.18% ± 4.46) compared with self-pacing (32.51% ± 5.07; *P* < 0.001, Cohen’s *d* = 1.117, large effect size).

In summary, the most pronounced reductions in heart rate variability with LED pacing were observed in the 0–50 m, 50–100 m, and the overall 200 m segments, where large or very large effect sizes were found. Moderate and small but still significant effects were observed in the 150–200 m and 100–150 m segments, respectively. These findings reflect enhanced physiological stability under LED pacing, particularly during the initial and middle phases, as well as over the entire trial (Table 6).

**TABLE 6:** Comparison of normalized HR range (CV, %) between control and LED pacing conditions across 200 m freestyle segments (n = 12).

Segment	Self-paced	LED pacing	*t*	*P*	95% CI	ES, *d*
0–50 m	7.60 ± 1.30	6.05 ± 0.98	5.034	<0.001	0.874∼2.231	1.345
50–100 m	8.58 ± 1.44	5.94 ± 0.89	8.838	<0.001	1.988∼3.306	2.204
100–150 m	7.85 ± 1.81	7.29 ± 1.57	4.239	0.001	0.267∼0.844	0.328
150–200 m	8.60 ± 1.55	7.57 ± 1.33	5.148	<0.001	0.591∼1.473	0.715
0–200 m (entire segment)	32.51 ± 5.07	27.18 ± 4.46	8.139	<0.001	3.893∼6.779	1.117

## 4 Discussion

This study systematically evaluated the effects of an intelligent LED pacing system on performance and physiological responses in 200 m freestyle swimming among trained athletes. After Bonferroni correction, LED pacing did not significantly improve total race time or split times, and pacing stability (CV for split times) also showed no significant difference between conditions. Notably, LED pacing also resulted in a smaller increase in blood lactate concentration from pre-to post-exercise, as well as lower heart rate range and heart rate CV%, reflecting improved metabolic and cardiovascular regulation. These results indicate that visual pacing feedback may affect both pacing execution and physiological regulation in trained swimmers.

Although nominal decreases in race time were observed across multiple segments, including the 50–150 m middle sections as well as the overall 200 m time, these changes were small in effect size and did not remain significant after Bonferroni correction. Similarly, the 0–50 m and 150–200 m segments showed no meaningful differences between conditions, further indicating that LED pacing exerted minimal impact on split performance throughout the race. Consistently, both the nominal improvements in total race time and the changes in split time CV% were small in magnitude and lost significance after correction, suggesting that for highly trained athletes with already refined pacing abilities, LED pacing provides little additional performance benefit and only limited improvements in pacing stability (Puce et al., 2022). Additionally, the brevity of the 200 m event, with only four splits available for CV% calculation, may have further reduced the sensitivity for detecting pacing changes (Lara and Del Coso, 2021). Previous research has shown that elite athletes often display ceiling effects, with training or ergogenic interventions producing only minimal performance gains in short or middle-distance events (Karabıyık et al., 2023; Cunha et al., 2019; Mujika et al., 1996). By contrast, pacing interventions such as external feedback or visual guidance appear to exert greater influence in longer-distance races or among athletes with less experience, where self-regulation is less optimized and external cues can enhance pacing consistency (Dobiasch et al., 2021; Menting et al., 2022; Konings and Hettinga, 2018). Such differences may reflect both the training status of the participants and the nature of the event: while elite swimmers in a 200 m trial have limited headroom for improvement, external pacing may be more effective in longer distances or in athletes with less optimized self-regulation.

While the direct performance effects of LED pacing were limited, the system nonetheless yielded distinct physiological benefits, as swimmers exhibited a significantly smaller rise in post-exercise blood lactate with a comparatively large effect size, despite faster overall times, making lactate regulation the most meaningful physiological outcome observed in this study. This outcome suggests that external pacing promotes a more economical use of anaerobic metabolism, which is particularly relevant to the 200 m freestyle where both aerobic and anaerobic pathways are heavily recruited (Massini et al., 2021). By constraining premature acceleration in the opening segments, LED guidance likely reduced early reliance on glycolysis, thereby delaying excessive lactate accumulation and attenuating the onset of intramuscular acidosis (Sengoku et al., 2024; Ruiz and Robazza, 2020). Such regulation of metabolic demand is important in middle-distance events, where an optimal balance between aerobic support and anaerobic contribution determines performance sustainability (Massini et al., 2021). Similar observations have been reported in interval training studies using external feedback, where mean lactate accumulation and variability in responses were both reduced (Bishop et al., 2002). Importantly, in shorter races such as the 200 m, pacing technology primarily acts to prevent early lactate surges (Morin and Samozino, 2016), whereas in longer events it helps conserve anaerobic reserves for greater late-race output (Quittmann et al., 2023). Thus, the effect of LED pacing on lactate dynamics is context-dependent, varying with race distance and energy system demands (Foster et al., 2023). In practice, such external guidance enables athletes to enhance performance while limiting avoidable metabolic stress.

The cardiovascular response also benefited from LED pacing, as indicated by reduced heart rate fluctuation and lower heart rate variability across all segments. Paced swimmers showed a smoother heart rate trajectory, reflecting steadier exertion without the abrupt surges that are common in self-paced efforts. These results are consistent with running studies and heart rate-guided protocols in other sports, where real-time feedback has been shown to stabilize internal load and enhance efficiency (Van Hooren et al., 2020). Lower heart rate variability during LED pacing likely reflects both improved physiological regulation and more consistent pacing behavior. One possible mechanism is that external pacing reduces the reliance on internal cues and decision-making, thereby minimizing anticipatory anxiety and unnecessary sympathetic activation, which in turn leads to lower overall cardiovascular strain during exercise (Zhao et al., 2024; Freudenheim et al., 2010). Although maximal effort naturally led to high end-race heart rates in both conditions, the LED pacing trials were characterized by smaller heart rate ranges and lower variability across segments. This indicates that the progression toward maximal exertion was more evenly regulated, with fewer abrupt surges in cardiovascular load. In practice, such steadier regulation may help swimmers sustain high-intensity sets with relatively lower physiological strain, which could facilitate recovery and promote more consistent performance across repeated training sessions (Neuloh et al., 2024; Sorgente et al., 2023; Oliveira et al., 2023). For coaches, these findings suggest that LED pacing might be strategically incorporated into workouts that emphasize mid-race speed maintenance or lactate tolerance, as it provides a consistent external cue to support controlled effort distribution (Szczepan and Zatoń, 2017; Neuloh et al., 2024; Fassone et al., 2025). Over time, repeated exposure to LED pacing may contribute to strengthening athletes’ pacing awareness, potentially enabling them to reproduce more effective race strategies even without external guidance (Fassone et al., 2025; Skorski et al., 2014; Hołub et al., 2023). Thus, beyond its immediate training applications, the system appears to offer coaches a practical means of structuring sessions more effectively and balancing physiological load, while also reinforcing the technical and psychological aspects of pacing (Karabıyık et al., 2023; Foster et al., 2023).

In this study, several paradoxical patterns were observed between performance and physiological indices, reflecting the complex relationship between external pacing and swimming outcomes. The most notable was in the 50–100 m segment, where HR CV showed the largest reduction while performance improvement was minimal, suggesting that enhanced physiological steadiness does not necessarily yield immediate time savings, especially when swimmers already approach an optimal cruising pace (Atlaoui et al., 2007; Flatt and Howells, 2019; McGibbon, 2020). Similarly, although blood lactate exhibited a large reduction, the concomitant decrease in total 200 m race time was small and failed to reach statistical significance, indicating that enhanced metabolic efficiency does not necessarily yield short-term performance improvements in highly trained athletes (Karabıyık et al., 2023; Eroglu et al., 2023). Finally, In the 0–50 m segment, a significant improvement in heart rate stability was observed, but this was not accompanied by a significant reduction in race time, consistent with the dominance of explosive start mechanics that constrain the impact of pacing cues (Morin and Samozino, 2016). Collectively, these paradoxes emphasize that the primary value of the LED pacing system in elite middle-distance swimming appears to be in refining physiological load management and energy allocation, rather than in fundamentally enhancing pacing uniformity or dramatically altering race strategies in highly trained athletes (Neuloh et al., 2024).

This study has several limitations that should be acknowledged. (Abbiss and Laursen, 2008). The investigation was restricted to a single 200 m event, which included only four split intervals and may limit sensitivity for detecting subtle differences in pacing variability. (Lara and Del Coso, 2021). The sample consisted of only 12 highly trained swimmers; while statistically justified, this relatively small and homogeneous group constrains the generalizability of the findings, particularly to less experienced or recreational athletes. (Fang et al., 2024). Although the 24-h interval between trials was considered reasonable for elite swimmers who routinely engage in daily high-intensity training, the possibility of residual fatigue and a learning effect due to repeated testing cannot be fully excluded. (Altavilla et al., 2018). The trial order was not randomized, and the improved outcomes observed under the LED pacing condition may have been partly influenced by a learning effect. (Szczepan and Zatoń, 2017). Participants had no prior exposure to LED pacing systems, which may have limited their ability to adapt to and fully utilize the visual pacing cues, potentially underestimating the system’s benefits. (Morais et al., 2022). The LED pacing protocol adopted a fixed pacing strategy that did not account for individual differences or permit adaptive adjustments during the swim. (Grannetia and Hoogkamer, 2024). Only acute effects were assessed, so the impact of long-term LED pacing training remains unclear. (Gonjo et al., 2021). Comprehensive physiological evaluation was limited, as the study did not integrate a broader range of physical or psychological assessments, such as perceived exertion or motivational factors. (Neuloh et al., 2024). The exclusive use of paired t-tests, which may not fully account for repeated measures or inter-individual variability. These limitations suggest that future studies should explore longer and multiple event distances, employ repeated and longitudinal interventions, recruit more diverse and larger cohorts, consider individualized or adaptive pacing strategies, and incorporate holistic assessments of both physiological and psychological parameters, as well as more nuanced pacing metrics such as intra-stroke velocity, acceleration, or biomechanical markers, to clarify the effects and applicability of LED pacing systems in swimming.

## 5 Conclusion

In highly trained swimmers, LED pacing produced limited effects on pacing consistency and overall race completion time but substantially reduced post-exercise blood lactate accumulation and heart rate fluctuations, indicating improved metabolic and cardiovascular efficiency. These findings suggest that visual feedback LED pacing system may hold greater value for managing internal load during training rather than providing immediate performance gains. Building on these findings, it would be valuable for future studies to examine how LED pacing systems can be applied in training to support performance improvements and ultimately enhance competitive performance.

## Data Availability

The original contributions presented in the study are included in the article/Supplementary Material, further inquiries can be directed to the corresponding authors.
