# Supplementary material for: Effects of a visual‐feedback LED pacing system in middle-distance pool freestyle swimming
Source: Front Bioeng Biotechnol. 2025 Oct 21;13:1679588. doi: 10.3389/fbioe.2025.1679588 (PMC12583020; doi:10.3389/fbioe.2025.1679588)
Supplement: Supplementary file 1 [file Table1.docx]

Supplementary Material

# Supplementary Tables

| Table 1 Baseline demographic and performance characteristics of participants (n = 12) | | | | | | |
| --- | --- | --- | --- | --- | --- | --- |
| Gender | Age (years) | Height (m) | Body mass (kg) | BMI (kg/m²) | Training experience (years) | Personal best 200 m freestyle pool time (s) |
| Female | 19.00 | 1.78 | 63.06 | 19.90 | 15.00 | 130.39 |
| Female | 20.00 | 1.73 | 65.34 | 21.83 | 15.00 | 141.75 |
| Female | 20.00 | 1.69 | 65.68 | 23.00 | 15.00 | 150.22 |
| Male | 23.00 | 1.85 | 67.88 | 19.83 | 16.00 | 123.06 |
| Male | 19.00 | 1.70 | 70.45 | 24.38 | 14.00 | 140.38 |
| Male | 21.00 | 1.78 | 75.52 | 23.84 | 15.00 | 142.96 |
| Female | 20.00 | 1.79 | 71.26 | 22.24 | 15.00 | 151.27 |
| Male | 22.00 | 1.82 | 70.25 | 21.21 | 15.00 | 125.76 |
| Male | 20.00 | 1.80 | 78.11 | 24.11 | 14.00 | 130.25 |
| Male | 20.00 | 1.72 | 65.37 | 22.10 | 16.00 | 140.73 |
| Female | 20.00 | 1.65 | 60.63 | 22.27 | 15.00 | 140.88 |
| Female | 20.00 | 1.65 | 55.46 | 20.37 | 13.00 | 135.46 |
